# Behaviour and molecular identification of *Anopheles* malaria vectors in Jayapura district, Papua province, Indonesia

**DOI:** 10.1186/s12936-016-1234-5

**Published:** 2016-04-08

**Authors:** Brandy St. Laurent, Sukowati Supratman, Puji Budi Setia Asih, David Bretz, John Mueller, Helen Catherine Miller, Amirullah Baharuddin, Shinta, Asik Surya, Michelle Ngai, Ferdinand Laihad, Din Syafruddin, William A. Hawley, Frank H. Collins, Neil F. Lobo

**Affiliations:** Eck Institute for Global Health, University of Notre Dame, Notre Dame, IN USA; National Institute of Health Research and Development, Ministry of Health, Jakarta, Indonesia; Eijkman Institute for Molecular Biology, Jakarta, Indonesia; University of Halueleo, Kendari, Indonesia; Department of Parasitology, Faculty of Medicine, Hasanuddin University, Makasaar, 90245 Indonesia; National Malaria Control Programme, Ministry of Health, Jakarta, Indonesia; UNICEF, Jakarta, Indonesia

**Keywords:** Malaria, *Anopheles*, Vector ecology, Molecular tools, Indonesia

## Abstract

**Background:**

Members of the *Anopheles punctulatus* group dominate Papua, Indonesia and Papua New Guinea (PNG), with a geographic range that extends south through Vanuatu. *An. farauti* and *An. punctulatus* are the presumed major vectors in this region. Although this group of species has been extensively studied in PNG and the southern archipelagoes within their range, their distribution, ecology and vector behaviours have not been well characterized in eastern Indonesia.

**Methods:**

Mosquitoes were collected in five villages in Jayapura province, Papua, Indonesia using human-landing collections, animal-baited tents and backpack aspirators. Mosquitoes were morphologically typed and then molecularly distinguished based on ribosomal ITS2 sequences and tested for *Plasmodium falciparum* and *P. vivax* infection using circumsporozoite ELISA and PCR.

**Results:**

The presence and vector status of *An. farauti4* in Papua, Indonesia is confirmed here for the first time. The data indicate that this species is entering houses at a rate that increases its potential to come into contact with humans and act as a major malaria vector. *An. farauti 4* was also abundant outdoors and biting humans during early evening hours. Other species collected in this area include *An. farauti 1*, *An. hinesorum*, *An. koliensis*, *An. punctulatus*, and *An. tessellatus.* Proboscis morphology was highly variable within each species, lending support to the notion that this characteristic is not a reliable indicator to distinguish species within the *An. punctulatus* group.

**Conclusions:**

The vector composition in Papua, Indonesia is consistent with certain northern areas of PNG, but the behaviours of anophelines sampled in this region, such as early and indoor human biting of *An. farauti 4*, may enable them to act as major vectors of malaria. Presumed major vectors *An. farauti* and *An. punctulatus* were not abundant among these samples. Morphological identification of anophelines in this sample was often inaccurate, highlighting the importance of using molecular analysis in conjunction with morphological investigations to update keys and training tools.

**Electronic supplementary material:**

The online version of this article (doi:10.1186/s12936-016-1234-5) contains supplementary material, which is available to authorized users.

## Background

The province of Papua, Indonesia has a high burden of malaria, with the highest reported malaria incidence among Indonesia’s 34 provinces. Both *Plasmodium falciparum* and *P. vivax* are prevalent in the province of Papua and throughout eastern Indonesia [1–3]. *P. vivax*, while often not a direct cause of mortality, largely contributes to the disease burden and morbidity in this region [4, 5]. The primary vectors of *P. vivax* are not well known, as mosquito surveys do not always include screening for *P. vivax* infection along with *P. falciparum.* While there have been many studies of the ecology of the *An. punctulatus* group in neighbouring Papua New Guinea (PNG), sampling of malaria vectors and characterization of their ecology and behaviour in Indonesian Papua is lacking. In particular, comprehensive morphological and molecular analysis of anophelines in this province is uncommon.

There are currently 13 recognized members of the *An. punctulatus* group and eight recognized members of the *An. farauti* complex [6–8]. Only *An. farauti 1* (hereafter referred to as *An. farauti*) and *An. punctulatus* are considered to be the primary malaria vectors. Some members of the *An. punctulatus* group can be distinguished through different morphological features, such as spotting on the palpi [9, 10], but the members of the *An. farauti* complex are morphologically indistinguishable and can only be typed to species using molecular tools [11]. There is significant morphological variation within even molecularly distinct species across their geographic range. There is also phenotypic variation within this complex, such as increased saline tolerance in *An. farauti* and *An. farauti 7* and large body size in *An. oreios* (formerly *An. farauti 6*) [12–14]. *An. farauti* is considered anthropophilic throughout its range and can exploit slightly saline larval habitats along the coast, unlike many other members of the *An. punctulatus* group [15]. Since most of these species have been shown to be capable of harbouring both *P. falciparum* and *P. vivax* parasites, the primary determinate of their status as a major vector of malaria is by their tendency to bite humans indoors or near human dwellings [16]. *An. farauti 4* (like most members of the *An. punctulatus* group) is known to be primarily outdoor-biting and outdoor-resting [17]. *An. farauti 4* and *An. hinesorum* (formerly *An. farauti 2*) have been found to have *P. falciparum* positivity rates comparable to *An. farauti* and *An. punctulatus* in Papua New Guinea [17, 18]. Species within the *An. punctulatus* group can also act as vectors of several different types of human filariasis [19].

Current malaria control efforts in Indonesia are primarily dependent upon the widespread use of long-lasting insecticide-treated bed nets (LLINs), which do not target outdoor-biting mosquito species [6, 20, 21]. Nonetheless, Indonesia Ministry of Health statistics show a marked decline in malaria incidence consequent to LLIN distribution and improved diagnosis and malaria case management over the past several years. A high prevalence of malaria was reported in this district in 2008 [22], and in 2014 was reported to have among the highest transmission rates in Indonesia (annual parasite incidence of 172 reported cases/1000 population in 2014) (Nyoman, Kabupatan, Jayapura, pers comm). Previous mosquito surveys in Papua, Indonesia, have not distinguished between cryptic members of the *An. farauti* complex [23] and most vector studies in this region pre-date the understanding of cryptic species complexes.

It is crucial to understand the bionomic traits of each vector species throughout their geographic range to be able to implement effective malaria control and elimination efforts, particularly since many of the species in the *An. punctulatus* group occur sympatrically [24]. This study adds to what is known about the *An. punctulatus* group within Indonesia through a brief survey and characterization of *Anopheles* species in five villages in Jayapura Province, Papua, Indonesia. The study illustrates the limits of reliance upon morphological identifications only for vector surveys in this particularly complex region. There are implications of the results for malaria control strategies in Indonesian Papua.

## Methods

### Site description

Papua, Indonesia is the easternmost province of Indonesia, bordering PNG. The eastern half of the island of Papua comprises PNG, while the western half comprises the Indonesian provinces of Papua and West Papua. The Malaria Transmission Consortium (MTC) collection sites in Papua province are in a series of villages along the coast near the provincial capital city Jayapura, very near to the border of PNG. Activities in this region include fishing, subsistence farming and harvesting spices in the jungle. Houses in this area are made of bamboo or thin pieces of wood with thatched roofs, many of which are up on stilts. Few houses have metal roofs. The open construction of these houses allows for mosquito entry from the bottom, sides and top. The health system in Papua is generally weaker than in other parts of Indonesia, which impacts surveillance, monitoring and treatment of malaria. Malaria transmission in this area is stable and high [3, 25].

**Fig. 1:**
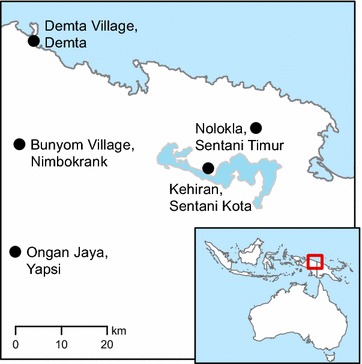
Map of Jayapura field collection sites. Malaria Transmission Consortium (MTC) sites where entomological collections took place. Adult mosquitoes were collected using backpack aspirators, human landing collections, and animal-baited tents in five villages both along the coast and inland. The *insert* shows the field collection area in relation to the rest of Indonesia and Australia

Mosquitoes were collected in five villages in Jayapura province, Papua, Indonesia during May of 2011: Demta village, Demta sub-district; Bunyom village, Nimbokrang sub-district; Kehiran village, Sentani Kota sub-district; Nolokla village, Sentani Timur (Harapan) sub-district, and Ongan Jaya village, Yapsi (Taja) sub-district (Fig. 1). The villages were spread across the district of Jayapura with Demta, Bunyom and Ongan Jaya villages being in forested areas close to rivers, while Kehiran and Nolokla villages were in agricultural areas.

### Mosquito collections

Several methods were utilized to collect anophelines. Backpack aspirators [26] were utilized hourly (from 18:00 to 06:00 hours) for 10 min to capture mosquitoes on bushes and foliage within and surrounding housing areas. Hourly human-landing catches (HLC) were performed both indoors and outdoors (from 18:00 to 06:00 hours). There were two indoor and two outdoor HLC collectors per night per site. Net tents were constructed using untreated netting with two wide openings situated around pig or cow baits. *Anopheles* mosquitoes were collected from the inside of the tent for ten min every hour (from 18:00 to 06:00 hours) using a mouth aspirator. The baited tents were situated away from human gathering areas and the human landing collectors. Collectors were advised not to wear any insect repellant during collections. Collections were performed for one night in each village except Kehiran, which had three nights of collections. All mosquitoes were collected, stored in holding cups labelled by hour until they were processed for morphological identification using keys for Indonesian anophelines [9]. High-quality adult specimens from the field collections were individually processed for detailed morphological descriptions of the palpi and proboscis, a key diagnostic feature of some members of the *An. punctulatus* group.

### Molecular processing of samples

Genomic DNA was isolated from individual specimens using a CTAB DNA extraction. Species were molecularly identified using sequences of the ribosomal DNA internal transcribed spacer region two (rDNA ITS2). This region of rDNA was isolated using PCR with ITS2A and ITS2B primers [27]. The amplified fragments were purified using an enzyme clean-up: 2U of Exonuclease 1 (USB Corporation, Cleveland, OH), 1U of Shrimp Alkaline Phosphatase (USB), and 1.8 μl of ddH20 were added to 8 μl of PCR product. This mixture was incubated at 37 °C for 15 min, followed by 15 min at 80 °C to inactivate the enzymes. PCR products were sequenced directly using Sanger sequencing on ABI 3730 xl DNA Analyzer platform (Applied Biosystems).

The ITS2 sequences were blasted against the NCBI database with BLASTn for confirmation of molecular species identification. Sequences were visually checked for sequence quality and potential contamination. Low quality or contaminated sequences were excluded from the analysis. Sequences with greater than 99 % sequence identity to voucher reference sequences were confirmed as that molecular species. Voucher sequences are available in the NCBI database for the species sampled in this study. The primary PCR diagnostics for these species is also based on the sequence of the rDNA ITS2 region.

The infection status of the mosquitoes was determined using the standard CDC sandwich CS-ELISA test for the detection of *P. falciparum*, *P. vivax*-210, and *P. vivax*-247 circumsporozoite (CS) proteins [28]. A sub-set of specimens were analysed for *Plasmodium* infection using a multiplex PCR for *P. falciparum* and *P. vivax* [29].

Statistical analyses to evaluate indoor and outdoor biting of *An. farauti* 4 and *An. koliensis* were performed using SPSS.

## Results

A total of 1071 (of 1968) individual mosquito specimens were successfully molecularly identified from Jayapura district, Papua province, Indonesia. Five morphological species were identified to six molecular species with an accuracy rate of 51 % (Table 1). These molecular species include *An. farauti, An. farauti 4, An. hinesorum, An. koliensis, An. punctulatus,* and *An. tessellatus* and were determined with sequence identity, greater than 99 % with reference sequences from voucher specimens.

**Table 1:** Molecular species identifications in underline with morphological species identification sub-heading by collection site

Molecular species ID	Village				
	Bunyom	Demta	Kehiran	Nolokla	Ongan Jaya
*An. farauti*					
*Morph ID: An. farauti s.l.*		*6*			
*An. farauti 4*					
*Morph ID: An. punctulatus*			*1*		
*An. farauti s.l.*	*6*		*424*	*5*	
*An. koliensis*			*112*		
*An. punctulatus*	*1*		*355*		
*An. hinesorum*					
*Morph ID: An. farauti s.l.*				*5*	*1*
*An. koliensis*					
*Morph ID: An. farauti s.l.*	*10*		*13*	*16*	*10*
*An. koliensis*			*17*	*3*	
*An. longirostris*		*1*			
*An. punctulatus*	*12*	*3*	*47*		*16*
*An. tesselatus*					*1*
*An. punctulatus*					
*Morph ID: An. punctulatus*					*3*
*An. tessellatus*					
*Morph ID: An. farauti s.l.*				*1*	
*An. koliensis*					*1*
*An. tesselatus*					*1*

Molecular identification based on ITS2 sequence, compared with field morphological identifications by site. There is a low concordance of morphological to molecular-level species identifications

There was a low level of accuracy when the specimens were identified morphologically in the field, with only 51 % accuracy using morphology alone. The few *An. farauti* and *An. punctulatus* specimens collected were morphologically identified accurately, while three specimens of the even more distinctive *An. tessellatus* was identified to three different species. *An. koliensis,* abundant in these collections and morphologically distinguishable from the other species, was identified as *An. koliensis* 13 % of the time. The most abundant species in the collection, *An. farauti 4,* was morphologically identified to *An. farauti s.l.* with 48 % accuracy. *An. koliensis* may be a species complex based on rDNA ITS2 sequence variation of samples found in other areas [18, 30], but this level of sequence variation was not observed within any of the molecularly identified specimens in this collection. These species have very similar and overlapping morphological characteristics, which can be further complicated by the quality of field-collected adult specimens. This level of accuracy in morphologically identifying field specimens when compared to molecular species identification is consistent with field collections in other areas of Southeast Asia and even countries within high transmission areas of Africa [31] (MTC data). This low level of morphological identification accuracy underlies the importance of incorporating molecular tools to help distinguish vector species.

**Fig. 2:**
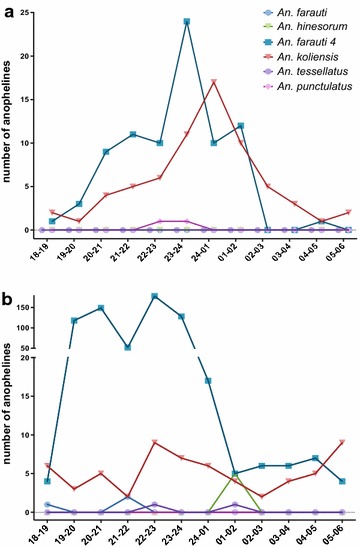
Biting times of species collected indoors (**a**), and outdoors (**b**). *An. farauti 4* is the predominant outdoor-biting species early in the evening. *An. farauti 4* and *An. koliensis* are the most abundant species sampled both indoors and outdoors. Note that *An. farauti* was not collected indoors

**Fig. 3:**
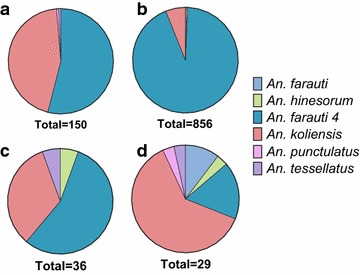
Molecularly confirmed species by sampling method. **a** HLC indoors, **b** HLC outdoors, **c** animal-baited traps, and **d** backpack aspiration of surrounding vegetation

The HLC collections indicate indoor and outdoor exposure of residents to host-seeking anophelines. Here, the majority of the species were collected both outdoors and early in the night, around dusk, at a time when people are extremely active, cooking and socializing outdoors. About 752 (74 %) of the total 1017 specimens collected were captured before midnight, with 394 (39 %) captured before 22.00. Only 299 (29 %) were caught between midnight and 06.00 (Fig. 2). This early evening biting behaviour is consistent with observations in PNG [18]. The total *Anopheles* species collected were more evenly distributed through the night. The *An. farauti 4* in this sample set were mostly collected in outdoor HLCs with 88 % of the total *An. farauti 4* collected captured in outdoor HLCs and 9 % in indoor HLCs. This species may be much more prone to biting humans than was previously suspected (Fig. 3). The second-most abundant species, *An. koliensis,* was captured primary in indoor HLCs, 45 and 35 % in outdoor HLCs. Backpack aspiration captured all six species sampled, but with relatively low numbers, and was not representative of human exposure to infectious bites when compared to indoor or outdoor HLCs (Fig. 2). Eighty percent of all of the *Anopheles* samples collected in this survey were captured in outdoor HLCs.

More than half of the specimens caught in indoor and outdoor HLCs, and animal-baited traps were *An. farauti 4* (Fig. 3). Fourteen percent of all of the anophelines were collected indoors, with the majority collected outdoors. *An. farauti 4* was 54 % of indoor HLCs, 93 % of outdoor HLCs, 56 % of animal-baited traps, and 17 % of backpack-aspirated mosquitoes from vegetation. *An. koliensis* was the second-most abundant species, representing 45, 6, and 33 % of indoor and outdoor HLCs, and animal baited tents, respectively. *An. koliensis* was the most abundant species captured in vegetation, representing 62 % of the catch. All other species were captured in very low abundance in each trap type. *An. farauti 1* was only collected outdoors, with six specimens captured in HLC and three in vegetation (Fig. 3).

To evaluate trap efficacy for the two most prevalent species in our collections, *An. farauti 4* and *An. koliensis*, multinomial regression was performed to compare these two species captured in indoor HLC, outdoor HLC, and backpack aspiration of surrounding foliage, which were performed every hour from 18:00 to 06:00 hours. The trap effect was not significant for *An. koliensis* (*p* = 0.021), and was significant for *An. farauti 4* (*p* ≤ 0.0001). *An. farauti 4* was more likely to be found outdoor HLC than indoor HLC, and more likely to be caught in indoor HLC than in backpack aspiration of vegetation. A binomial logistic regression of these same two species was performed to compare their propensity to be captured indoors vs. outdoors. *An. farauti 4* was 9.85 times more likely to be found outdoors than *An. koliensis.* Hourly biting profiles of *An. farauti 4* were compared using a Chi square goodness of fit test. More *An. farauti 4* were caught indoors before midnight than between the hours of midnight and 6 am (χ^2^: 15.12, df = 1, p = 0.0001). The number of *An. farauti 4* caught indoors was significantly higher between 23 and 24 h when compared to other hours (χ^2^ = 48.418; df = 1, p < 0.0001) (Additional file 1). The frequency of *An. farauti 4* outdoors before midnight was higher than between the hours of midnight and 6 am (χ^2^: 392.38, df = 1, p = <0.0001).

Four of the species collected in this survey are considered to be zoophilic, *An. hinesorum, An. farauti 4, An. koliensis,* and *An. tessellatus* and they were all captured in the animal-baited tents (Fig. 3). Animal-baited tents captured only 3 % of the total anophelines. Two of the four total *An. tessellatus* samples collected were in the animal-baited tents. Only 2 and 8 % of the total *An. farauti 4,* and *An. koliensis* specimens were captured in the animal-baited tents, with the vast majority captured in HLCs, indicating that both of these species have a higher human-biting preference than previously thought in this region. There were village-specific effects of species distribution and biting rates (Additional file 2). Futher sampling must be done to determine location-specific effects on species catch.

Human-biting rates were exceptionally high in Kehiran I village with about 84 bites per person per night outdoors and about 19 indoors. All sporozoite-positive mosquitoes were captured in this village, as were 98.7 % of *An. farauti 4* and 51.7 % of *An koliensis* captured. Four individual specimens from these collections were found to be carrying *P. vivax* sporozoites, three *An. farauti 4*, and one *An. koliensis* (Table 2). In this region, *An. farauti* and *An. punctulatus* are considered to be the primary vectors [8, 17, 32]. These two vector species represented a very small portion of the collection, only 0.8 % of the total collection. The majority of specimens collected were *An. farauti 4*, representing 89 % of the total collection and also found to be sporozoite positive for *P. vivax*. The three *P. vivax*-positive *An. farauti 4* and one *An. koliensis* were collected in outdoor HLCs (Table 2), with 0.4 and 1.6 % positivity rates, respectively.

**Table 2:** Individual specimens positive for *Plasmodium vivax*

Molecular ID	Morphological ID	Site collected	Method of collection	Hour of collection	Method of detection
*An. farauti* *4*	*An. punctulatus*	Kehiran	Outdoor HLC	22–23	PCR
*An. farauti* *4*	*An. farauti s.l.*	Kehiran	Outdoor HLC	23–24	ELISA Pv-210
*An. farauti* *4*	*An. farauti s.l.*	Kehiran	Outdoor HLC	20–21	ELISA Pv-210
*An. koliensis*	*An. koliensis*	Kehiran	Outdoor HLC	04–05	ELISA Pv-210

The species identification, collection information and method of *Plasmodium* detection are listed for individual mosquito specimens found positive for *P. vivax.* Note that *An. farauti 4* and *An. koliensis*, two species not considered primary vectors, are positive for *P. vivax.* These four positive samples represent a 0.6 % *P. vivax* positivity rate of the outdoor HLC collections in Kehiran village

The molecularly confirmed *An. farauti 4* specimens were identified morphologically as *An. punctulatus* 39 % of the time (Table 1). This is an issue for both species identification and the allocation of vector bionomic traits to the appropriate species. In this case, without molecular species identification, the transmission of malaria and early biting behaviour by *An. farauti 4* would be attributed to *An. farauti s.l.* and *An. punctulatus. An. opheles farauti 4* is not considered to be a major malaria vector, although it has been shown to have high *P. falciparum* and *P.vivax* infection rates during mosquito surveys in PNG [17, 33] and has also been identified to be extremely polymorphic for proboscis characteristics which differentiate members of the *An. punctulatus* group [8]. Although these species have some overlapping morphological and behavioural traits, they are evolutionarily distinct [24].

Members of the *An. punctulatus* group have very similar morphological characteristics, often only being distinguishable by banding patterns on the underside of the proboscis, a key morphological characteristic for this group [8, 9]. Even within these collections in Papua, Indonesia, additional morphological variants of these proboscis types were identified (Table 3; Additional file 3). Detailed morphology of the proboscis of individual specimens revealed morphological variation, even within cryptic species [8]. This high level of morphological variation within species has been observed elsewhere within a single cryptic species in Indonesia, such as *An. epiroticus* and *An. vagus* (S Zubaidah, unpublished). The *An. farauti 4* specimens in the collections were particularly morphologically variable for this trait, with specimens with each well-known phenotype, and several new phenotypes, represented within this species in the collection (Table 3; Additional file 3) [34]. *An. farauti* and *An. punctulatus* had the expected A1 and B phenotypes, respectively, while each other species had unexpected or mixed phenotypes. The assortment of proboscis phenotypes, within even cryptic species, supports the need for molecular tools to distinguish all species in the *An. punctulatus* group, particularly in areas where these species occur in sympatry.

**Table 3:** Variation of morphological proboscis phenotypes within molecular species

Species	Proboscis morphology type														
	A1	B2	B3	C3	C4	C5	C6	C7	C8	C9	C10	C11	C12	C13	C14
*An. farauti*	6														
*An. farauti 4*	408	85	188	2	66	15	1	4	16	25	11	63	2	10	4
*An. hinesorum*	2	1							2		1				
*An. koliensis*	12	80	4		32			2	3	3	1	2	8		
*An. punctulatus*		1	2												
*An. tessellatus*	2														

A1–C12 Phenotypic proboscis characteristics of the *An. punctulatus* group (see additional file for sketch) after Rozebloom and Knight [34] and Bryan [36]. A1 represents *An. farauti* and *An. koliensis* characteristics, B1–2 represents typically *An. punctulatus* features, and C phenotypes are represented within different species of the group. C13 represents a new phenotype (with a small pale spot behind labellum located in the middle of proboscis and uniform colouring of the wing sub-costa), C14 proboscis phenotypes do not fit into the other categories

## Conclusions

Results of this survey illustrate the necessity for use of molecular tools to ensure accurate species identification in Indonesian Papua. Morphological identification of these specimens was wrong nearly as often it was correct, highlighting the need for supplementation of morphological keys with molecular information to allow production of better training tools for morphological identification. Morphologically based routine mosquito surveillance is needed and inevitable, but updated keys with periodic integration of molecular identification would minimize inaccuracies. There are an increasing number of species diagnostic tools that are decreasing in cost per sample, including multiplex PCRs, to identify members of the *An. punctulatus* group. Tools such as these should be incorporated into malaria control programmes in this region to accurately identify vector species and associate them with their specific bionomic traits in order to efficiently target them, particularly in an area with such high transmission rates [6, 25, 35]. Without distinguishing field-collected specimens at a molecular level, the indoor and human-biting behaviours of *An. farauti 4* and *An. koliensis* found in this study would have been attributed to *An. farauti s.l.* and assumed to be *An. farauti*.

Although *An. farauti 4* and *An. koliensis* were implicated as malaria vectors in this survey, it remains unclear to what extent this result can be generalized. Samples were collected in 1 month (May 2011) and nearly all of the *An. farauti 4* specimens came from a single village. Although this data concludes that this species transmits malaria in Papua province, Indonesia, it is not yet know how widely distributed the species is in Indonesian Papua, or even in Jayapura district. Further studies will be needed to know if the species is seasonally abundant or regularly infected, and information on its larval habitats is lacking. It is unknown whether a change in species abundance of different anophelines may have occurred in Papua subsequent to massive scale-up of malaria control interventions targeting indoor-biting species. Such information would allow the National Malaria Control Programme to make confident inferences as to which interventions might be most effective in the future in this last redoubt of malaria in Indonesia.

## Additional files

10.1186/s12936-016-1234-5 The frequency of *Anopheles farauti 4* mosquitoes caught outdoors. Chi square goodness of fit test of hourly biting rates of *An. farauti 4.* The frequency of *An. farauti 4* outdoors before midnight was higher than between the hours of midnight and 6 (χ^2^: 392.38, df = 1, p ≦ 0.0001). Bars show differences in hourly biting where ***p < 0.0001; **p < 0.001; *p < 0.05.
10.1186/s12936-016-1234-5 Hourly collection of molecular species by collection method: Indoor HLC, Outdoor HLC, Animal-baited tent, and Backpack aspiration in each of 5 villages in Papua, Indonesia. *Kehiran I collections represent 3 nights of collection while all other villages took place over a single night.
10.1186/s12936-016-1234-5 Variation on of morphological proboscis phenotypes of the *An. punctulatus* group A1-C12 Phenotypic proboscis characteristics of the *An. punctulatus* group adapted from Rozebloom and Knight [34] and Bryan [36] with an additional phenotype C-13.
