# Supplementary material for: Behaviour and molecular identification of Anopheles malaria vectors in Jayapura district, Papua province, Indonesia
Source: Malar J. 2016 Apr 8;15:192. doi: 10.1186/s12936-016-1234-5 (PMC4826537; doi:10.1186/s12936-016-1234-5)
Supplement: Supplementary file 1 — 10.1186/s12936-016-1234-5 The frequency of Anopheles farauti 4 mosquitoes caught outdoors. Chi square goodness of fit test of hourly biting rates of An. farauti 4. The frequency of An. farauti 4 outdoors before midnight was higher than between the hours of midnight and 6 (χ2: 392.38, df = 1, p ≦ 0.0001). Bars show differences in hourly biting where ***p < 0.0001; **p < 0.001; *p < 0.05. [file 12936_2016_1234_MOESM1_ESM.docx]

No. *An. farauti 4* collected

Additional file 1. **The frequency of *Anopheles farauti 4* mosquitoes caught outdoors.** Chi-square goodness of fit test of hourly biting rates of *An. farauti 4.* The frequency of *An. farauti 4* outdoors before midnight was higher than between the hours of midnight and 6 (χ^2^: 392.38, df = 1, p=<0.0001). Bars show differences in hourly biting where *** p < 0.0001; ** p < 0.001; * p < 0.05.
