# Supplementary material for: Behaviour and molecular identification of Anopheles malaria vectors in Jayapura district, Papua province, Indonesia
Source: Malar J. 2016 Apr 8;15:192. doi: 10.1186/s12936-016-1234-5 (PMC4826537; doi:10.1186/s12936-016-1234-5)
Supplement: Supplementary file 2 — 10.1186/s12936-016-1234-5 Hourly collection of molecular species by collection method: Indoor HLC, Outdoor HLC, Animal-baited tent, and Backpack aspiration in each of 5 villages in Papua, Indonesia. *Kehiran I collections represent 3 nights of collection while all other villages took place over a single night. [file 12936_2016_1234_MOESM2_ESM.pdf]

**Trap type**<br>**Village**<br>Species	Hour of collection										
**Indoor HLC**	18-19	19-20	20-21	21-22	22-23	23-24	24-01	01-02	02-03	03-04	04-05	05-06
**Bunyom**												
An. farauti 4					1							
An. koliensis	1		2	1	1		2	1				1
**Kehiran I***												
An. farauti 4	1	3	9	11	9	24	10	12			1	
An. koliensis	1		1	4	5	7	14	9	2	1	1	1
**Nolokla**												
An. koliensis						4			3	1		
**Ongan Jaya**												
An. koliensis		1	1				1			1		
An. punctulatus						1	1					
**Outdoor HLC**	18-19	19-20	20-21	21-22	22-23	23-24	24-01	01-02	02-03	03-04	04-05	05-06
**Bunyom**												
An. farauti 4			1	1								
An. koliensis	2				1						1	1
**Demta**												
An. farauti 1	1			2								
**Kehiran I***												
An. farauti 4	4	115	142	49	177	128	17	4	4	2	5	3
An. koliensis			3		7	5	3	1			3	7
**Nolokla**												
An. farauti 4									1	1		
An. hinesorum								3				
An. koliensis									1	3		
**Ongan Jaya**												
An. koliensis	2	2	1	1	1	2		1	1	1		
**Animal-baited net**	18-19	19-20	20-21	21-22	22-23	23-24	24-01	01-02	02-03	03-04	04-05	05-06
**Bunyom**												
An. farauti 4		1										
An. koliensis	2											
**Kehiran I***												
An. farauti 4		2	6	2	1				1	3	2	1
**Nolokla**							3	6			1	
An. farauti 4								1				
An. hinesorum								2				
An. koliensis							3	2			1	
An. tessellatus								1				
**Ongan Jaya**												
An. koliensis		1	1	1								1
An. tessellatus					1							
**Backpack aspiration of vegetation**	18-19	19-20	20-21	21-22	22-23	23-24	24-01	01-02	02-03	03-04	04-05	05-06
**Bunyom**												
An. farauti 4			1	1	1							
An. koliensis	2		1	1			1				1	
**Demta**												
An. farauti 1	1	1										1
An. koliensis				1					3			
**Nolokla**	1			1	1							
An. farauti 4	1			1								
An. koliensis					1							
**Ongan Jaya**												
An. hinesorum	1											
An. koliensis		2	1		1					1	1	1
An. punctulatus										1		
An. tessellatus	1											

Additional File. Table.

Hourly collection of molecular species by collection method: Indoor HLC, Outdoor HLC, Animal-baited tent, and Backpack aspiration in each of 5 villages in Papua, Indonesia. *Kehiran I collections represent 3 nights of collection while all other villages took place over a single night
