# Supplementary material for: Behaviour and molecular identification of Anopheles malaria vectors in Jayapura district, Papua province, Indonesia
Source: Malar J. 2016 Apr 8;15:192. doi: 10.1186/s12936-016-1234-5 (PMC4826537; doi:10.1186/s12936-016-1234-5)
Supplement: Supplementary file 3 — 10.1186/s12936-016-1234-5 Variation on of morphological proboscis phenotypes of the An. punctulatus group A1-C12 Phenotypic proboscis characteristics of the An. punctulatus group adapted from Rozebloom and Knight [34] and Bryan [36] with an additional phenotype C-13. [file 12936_2016_1234_MOESM3_ESM.pdf]

Proboscis morphotypes

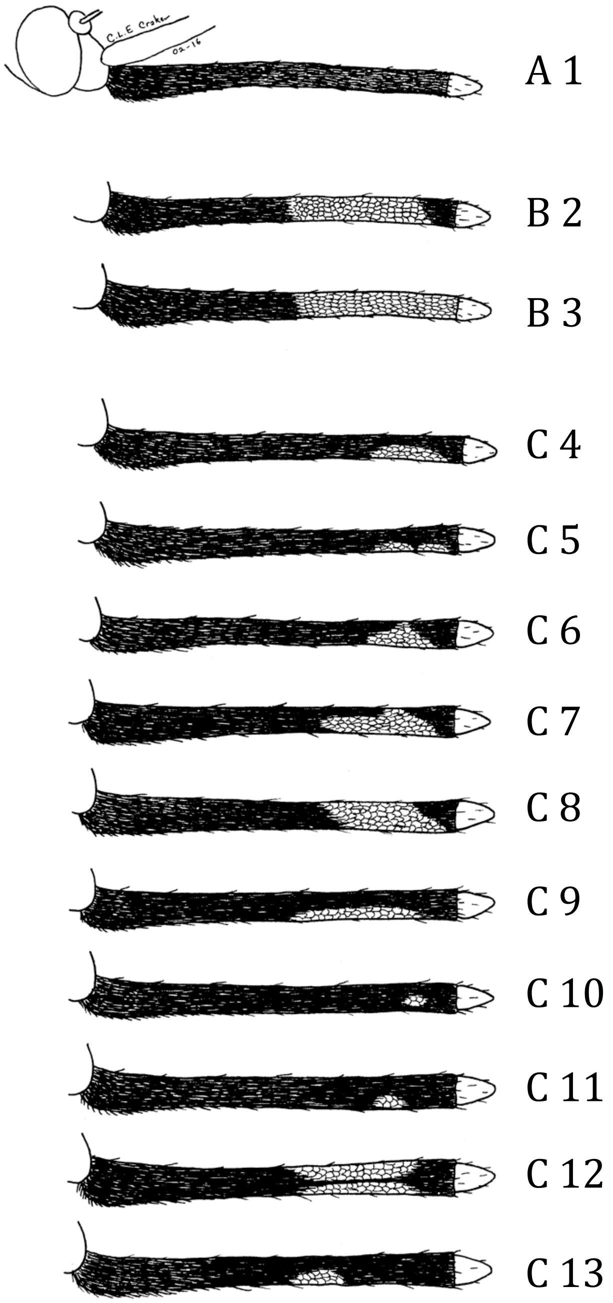

Additional figure. **Variation of morphological proboscis phenotypes of the *An. punctulatus* group**
A1-C12 Phenotypic proboscis characteristics of the *An. punctulatus* group adapted from Rozebloom and Knight 1946 [34] and Bryan 1974 [36] with an additional phenotype C-13.
